# Voxel-based analysis unveils regional dose differences associated with radiation-induced morbidity in head and neck cancer patients

**DOI:** 10.1038/s41598-017-07586-x

**Published:** 2017-08-03

**Authors:** Serena Monti, Giuseppe Palma, Vittoria D’Avino, Marianna Gerardi, Giulia Marvaso, Delia Ciardo, Roberto Pacelli, Barbara A. Jereczek-Fossa, Daniela Alterio, Laura Cella

**Affiliations:** 1 IRCCS SDN, Naples, Italy; 2 Institute of Biostructures and Bioimaging, National Research Council (CNR), Naples, Italy; 3 Department of Radiotherapy, European Institute of Oncology, Milan, Italy; 4 Department of Advanced Biomedical Sciences, Federico II University School of Medicine, Naples, Italy; 5 Department of Oncology and Hemato-oncology, University of Milan, Milano, Italy

## Abstract

The risk of radiation-induced toxicity in patients treated for head and neck (HN) cancer with radiation therapy (RT) is traditionally estimated by condensing the 3D dose distribution into a monodimensional cumulative dose-volume histogram which disregards information on dose localization. We hypothesized that a voxel-based approach would identify correlations between radiation-induced morbidity and local dose release, thus providing a new insight into spatial signature of radiation sensitivity in composite regions like the HN district. This methodology was applied to a cohort of HN cancer patients treated with RT at risk of radiation-induced acute dysphagia (RIAD). We implemented an inter-patient elastic image registration framework that proved robust enough to match even the most elusive HN structures and to provide accurate dose warping. A voxel-based statistical analysis was then performed to test regional dosimetric differences between patients with and without RIAD. We identified a significantly higher dose delivered to RIAD patients in two voxel clusters in correspondence of the cricopharyngeus muscle and cervical esophagus. Our study goes beyond the well-established organ-based philosophy exploring the relationship between radiation-induced morbidity and local dose differences in the HN region. This approach is generally applicable to different HN toxicity endpoints and is not specific to RIAD.

## Introduction

In radiation therapy (RT) treatment planning (TP), the dose-volume histogram (DVH), introduced in the late 1970s^1^, has proved to be a valuable tool for summarizing a three-dimensional (3D) dose distribution in a two-dimensional (2D) graph. For practical reasons, most biological models developed in RT to predict tumor control or normal tissue side effects start with the target or organ-at-risk (OAR) DVH, respectively, rather than with the complete radiation dose distribution. This implies that the structures of interest are considered homogeneous, and, therefore, any spatial dose distribution information and possible inhomogeneity in regional organ radiosensitivity are disregarded. Also, different definition of the OARs may influence the toxicity prediction^2^.

After nearly 40 years, we have reached the maturity and computational tools to exploit the information included in the full 3D dose distribution. Recently, 2D or 3D methods for dose distribution analysis (dose mapping), collectively referred to as voxel-based (VB) methods, have tried to evaluate local dose response patterns beyond the organ-based philosophy, and have been proposed as alternative approaches to the DVH for identifying dose sensitive regions of normal tissue. These methods have been successfully applied to prostate and thoracic cancer patients^3–8^, but, to the best of our knowledge, no VB analysis has been performed on patients with tumors in the head and neck (HN) area.

RT is one of the most effective modalities for the treatment of HN cancers. However, because of the complex shape of target volumes in close proximity to sensitive organs, it may be associated with acute and late radiation morbidities such as xerostomia, mucositis and dysphagia affecting the patient’s quality of life^9^. Many studies have investigated dose-volume predictors as well as spatial dose metrics in order to minimize those radiation induced side effects^10–14^. However, many of these studies might be limited by a large spectrum of issues ranging from the difficulty or inconsistency in the delineation of the related OARs to the use of a single summary measure from the DVH. In particular, the definition and contouring of swallowing-related normal tissues (i.e., the pharyngeal constrictor muscles, base of tongue, supraglottic larynx, soft palate, cricopharyngeal muscle and cervical esophagus) can be time consuming and prone to inter- and intra-operator variations^13, 15^.

At the same time, the use of the intensity-modulated RT (IMRT) for HN cancer treatment as a standard technique calls for an afterthought in the definition of dose and volume constraints for plan optimization in order to take full advantage of IMRT improved therapeutic ratio. This issue is even more important in light of the recent introduction in TP systems of automated engines designed to create an optimized plan with minimal user interaction. In HN region, in particular, they proved able to further improve the clinical quality of IMRT plans and OAR sparing^16, 17^.

Given this background, the current study was designed to apply a VB analysis in order to explore the correlation between radiation induced acute dysphagia (RIAD) and local dose differences in a cohort of patients treated with RT for HN cancer. The VB approach relies on mapping all patient dose distributions into a single reference case anatomy, which serves as anchor for local dosimetric evaluations. Compared to other districts, the HN presents peculiar difficulties in the registration procedure, thus posing several challenges for the achievement of an accurate dose warping^18, 19^. We implemented an inter-patient elastic image registration (EIR) framework that proved robust enough to match even the most elusive structures involved in the investigated toxicity endpoint. Thereafter, a VB statistical analysis was performed to test dosimetric regional differences between patients with different outcomes. Finally, the application of the VB analysis in HN clinical TP optimization was verified.

## Materials and Methods

### Patient dataset

The patients analyzed in this study are part of a cohort of 100 subjects treated at the Department of Radiation Therapy of the European Institute of Oncology (Milano) for HN tumors, and prospectively evaluated for radiation-induced acute dysphagia (RIAD). The study was part of a research regarding image guided RT for HN cancer approved by the Ethical Committee of the European Institute of Oncology (Notification n. 94/11). All patients gave written informed consent. All experimental protocols and procedures were performed in accordance with the guidelines of the European Institute of Oncology. All clinical imaging data were used in anonymized form. Applied selection criteria included:

- Curative RT for HN cancer associated or not with concurrent or induction chemotherapy;
- No prior surgery in HN region;
- No baseline mechanical dysphagia;
- No clinically relevant weight loss in the 3 months prior to RT;
- No requirement of enteral nutrition before RT.

A subset of 42 patients resulted eligible for the analysis. Twenty-three of these were treated with 3D conformal RT and the remaining 19 patients with IMRT. TP Computer Tomographic (CT) scans were acquired with an in-plane matrix of 512 × 512 and a slice thickness of 3 mm. Standard OARs, including oral cavity, larynx and esophagus for HN, were contoured according to RTOG 0615^20^. Median total prescribed RT dose was 70 Gy (range: [64, 70] Gy) in daily fractions of 2 Gy. All patients but one received induction (6/42) or concurrent chemotherapy (35/42).

Nine patients developed a severe RIAD (CTCAE v.4.0 grade ≥3). Patient, disease and treatment-related characteristics were examined according to the development of RIAD by univariate statistical methods: categorical variables were tested by Pearson’s χ^2^-test or Fisher’s exact test when appropriate; continuous variables were tested by Mann-Whitney U-test. None of the considered clinical variables (gender, age, tumor stage and location, RT technique and chemotherapy) showed a significant correlation with RIAD. A detailed description of the patient and treatment characteristics has been reported elsewhere (submitted).

### Data processing

Individual DICOM-RT plans (CT scans, dose maps, and contoured structures) were converted into a Matlab (MathWorks, Natick, MA) object using the Computational Environment for Radiation therapy Research software^21^.

The VB analysis, which entails

**Figure 1:**
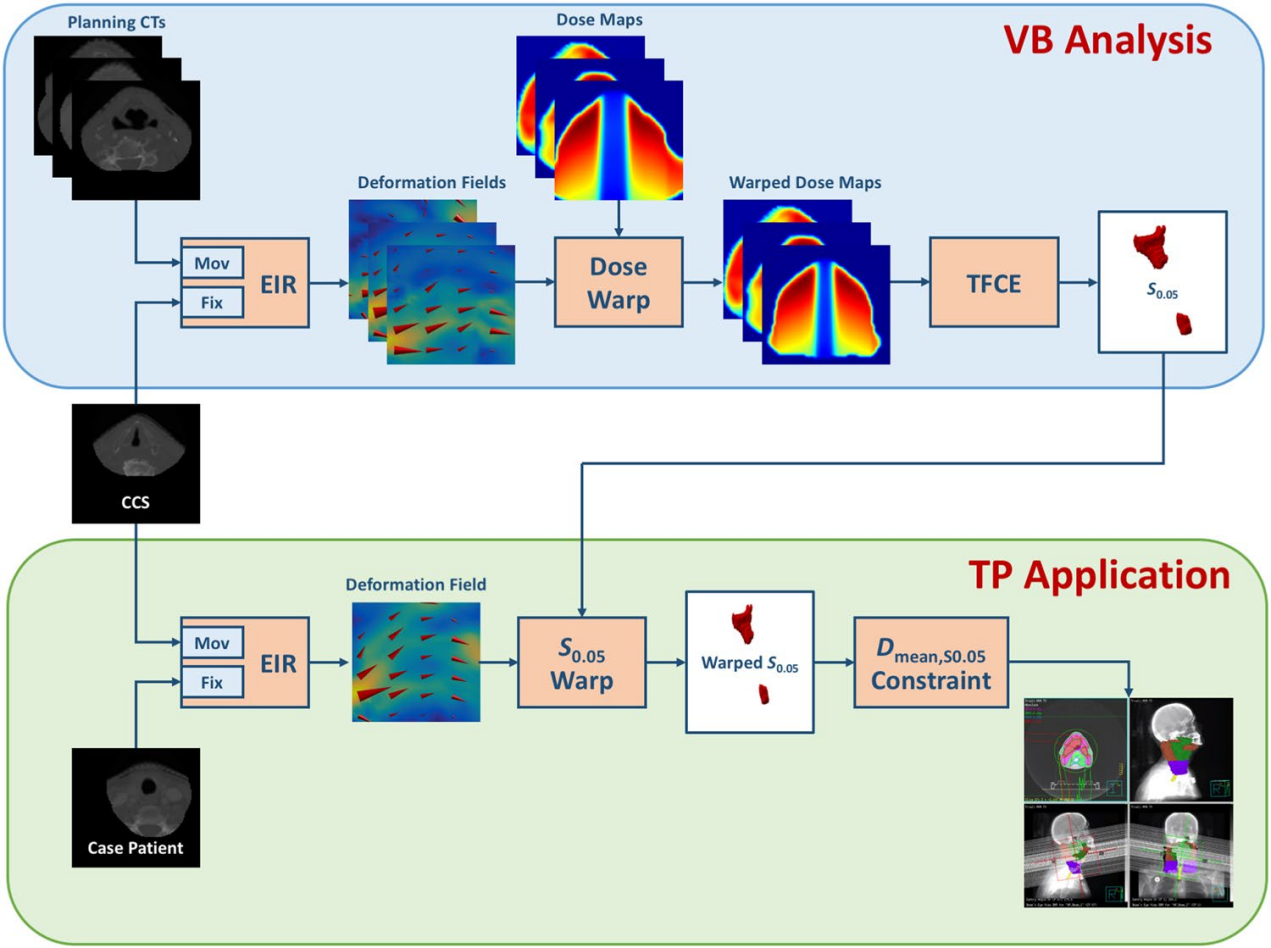
Schematic flowchart of the voxel-based (VB) analysis (upper panel) and of its treatment planning (TP) application (lower panel) in head and neck (HN) cancer patients. Planning CTs are registered to the common coordinate system (CCS) by an elastic image registration (EIR) tool. The obtained deformation fields are used to warp the dose maps into the CCS. A statistical mapping scheme (TFCE) is applied to extract the clusters of voxels (*S*_0.05_) significantly associated with higher doses released in patients developing the radiation-induced toxicity. For any new HN patient (case patient), the *S*_0.05_ region is warped as a new avoidance structure into the DICOM-RT object according to the deformation field that maps the CCS into the considered planning CT, and the corresponding mean dose (D_mean,*S*0.05_) is used as planning constraint.

1. inter-patient normalization consisting in the registration of CT and dose for each patient to a common spatial reference
2. statistical analysis of regional dose differences between RIAD and non-RIAD patients’ groups,

was performed according to the scheme developed in Palma *et al*.^5^, as illustrated in the upper panel of Fig. 1.

Briefly, a masked planning CT of each patient was registered to a common coordinate system (CCS) via a log-diffeomorphic demons approach^22^, an EIR tool that guarantees the invertibility of the deformation field. The mask was given by the intersection of the patient’s body structure with the dilation (by a spherical structuring element of 30 mm radius) of the union of oral cavity, larynx and esophagus. The obtained deformation fields were used to map the dose of each patient into the CCS. The dose warping accuracy was evaluated by Dice^23^, modified Hausdorff distance (MHD)^24^ and dose-organ overlap (DOO)^3^ scores.

Next, a preliminary significance analysis of the dose differences between groups was performed according to a non-parametric multiple comparison permutation test (10^4^ permutations) by single maximum threshold, generally referred to as the normalized maximum dose difference, *T*_max_^25^. Having obtained the evidence that a statistical regional difference holds, a statistical mapping scheme for non-parametric multiple permutation inference on dose maps with threshold-free cluster enhancement (TFCE)^26^ was applied in order to highlight the clusters of voxels significantly associated with higher doses released in RIAD patients, within the region of the swallowing related structures (superior, middle and inferior pharyngeal constrictor muscles, cricopharyngeal muscle, cervical esophagus, base of tongue, supraglottic and glottic larynx^15^) segmented in the CCS.

A set of subregions {*S*_*p*_} was thus defined as the family of the sublevel sets of the significance map in correspondence of the TFCE significance level *p*. A Receiver Operating Characteristic- (ROC) based test was performed on the mean doses {*D*_*m,i*_|*i* = 1, …, 42} extracted from *S*_0.05_. Furthermore, the significance (*p*_*U*_) of the mean dose differences on *S*_*p*_ between RIAD and non-RIAD patients was evaluated by a Mann-Whitney *U* test and plotted as function of *p*.

### Treatment Planning application

In order to demonstrate the practical relevance of the above analysis, a new IMRT plan (Plan_1_) for an oropharyngeal cancer patient (not included within the analyzed cohort) was generated according to Merlotti *et al*.^27^ for dose prescription, OARs dose-volume objectives and plan optimization. Three dose levels were prescribed to planning target volumes (PTVs): 70 Gy (2 Gy/die) for macroscopic disease (PTV1), 63 Gy (1.8 Gy/die) for high risk areas (PTV2), and 58.1 Gy (1.66 Gy/die) for low risk areas (PTV3).

A second plan (Plan_2_) was generated as a re-optimization of Plan_1_ by using the *S*_0.05_ structure as an avoidance region. For this purpose, the *S*_0.05_ region was warped as a new structure into the patient DICOM-RT plan according to the deformation field that maps the CCS into the considered planning CT reference, estimated as before via log diffeomorphic demons. For the *S*_0.05_ structure we imposed the constraint that the corresponding mean dose (D_mean,*S*0.05_) would not exceed the first percentile on RIAD patients (lower panel of Fig. 1). In this regard, since the number of RIAD patients is relatively low (in particular, less than 100), the first percentile was estimated as

1 $$\mathrm{mean}(\{{D}_{m,i}|i\in {RIAD}\})+\mathrm{stddev}(\{{D}_{m,i}|i\in {RIAD}\})\cdot \mathrm{probit}\,0.01$$

provided that the Kolmogorov-Smirnov test on $\{{D}_{m,i}|i\in {RIAD}\}$ indicates that Gaussianity held.

Both plans were implemented using Volumetric Modulated Arc Therapy (VMAT) technique with two 360°-arcs and nominal energy of 6MV. Plans were optimized by the automatic optimization module integrated in the TP system Pinnacle 9.10 (Philips Radiation Oncology Systems, Fitchburg, WI).

### Data availability

The data analyzed in the present study are available at http://www.ibb.cnr.it/?command=viewcms&and2=80&id=187.

## Results

**Figure 2:**
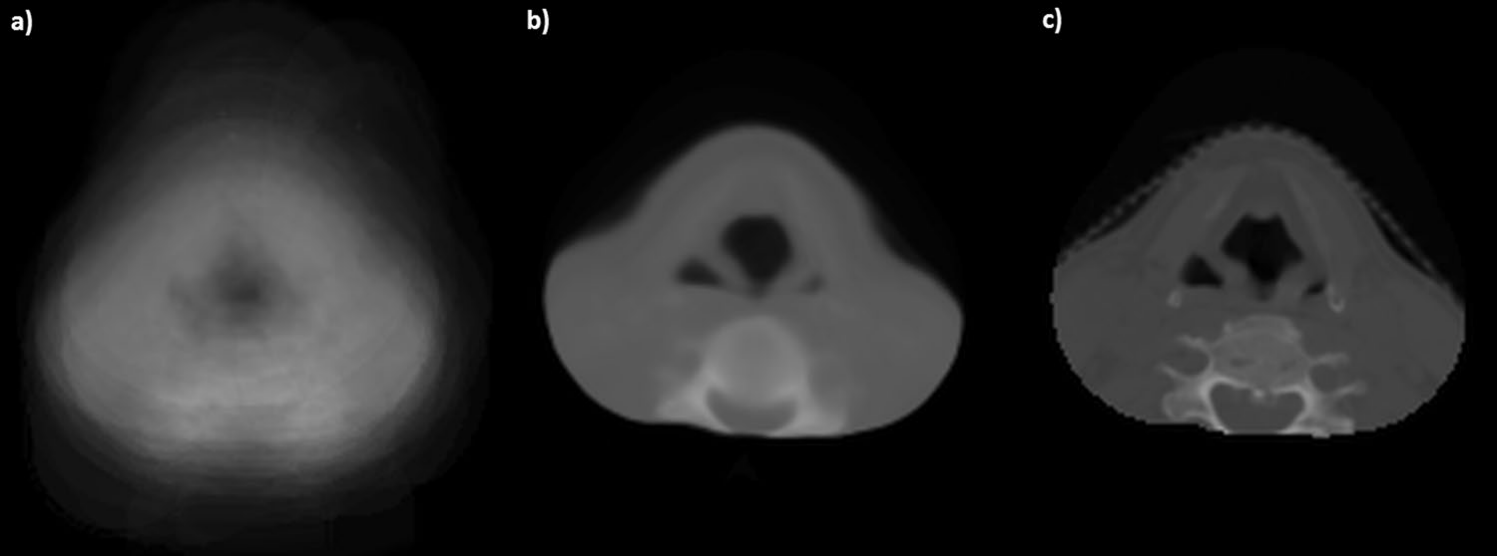
Elastic image registration (EIR) results. Average of the patient population computed tomographic scans (**a**) before EIR and (**b**) after EIR, compared to (**c**) the corresponding reference patient’s scan.

The registration process resulted in a geometrically robust and accurate dose warping, as confirmed by visual inspection on CT images (Fig. 2). Indeed, the voxelwise average of the patients’ CTs resulted much sharper after EIR, compared to the blurred appearance of the mean original CT. Moreover, the comparison of medians and ranges of Dice, MHD and DOO scores, computed before and after EIR on the whole patient dataset, showed a consistent significant improvement of inter-patient match accuracy (Table 1).

**Table 1:** Registration scores for pre- and post-elastic image registration.

	Dice		MHD (mm)		DOO	
	pre	post	pre	post	pre	post
Median	0.67	0.80	2.31	1.22	0.51	0.66
Range	[0.03,0.83]	[0.59,0.82]	[0.53,24.13]	[0.78,4.46]	[0.02,0.68]	[0.46,0.76]
*p*-value^§^	<10^−5^		<10^−4^		<10^−6^	

*Abbreviations*: MHD, Modified Housdorff Distance; DOO, Dose-Organ Overlap. ^§^ *p*-values express the significance of the interpatient match improvement at Wilcoxon signed rank test.

**Figure 3:**
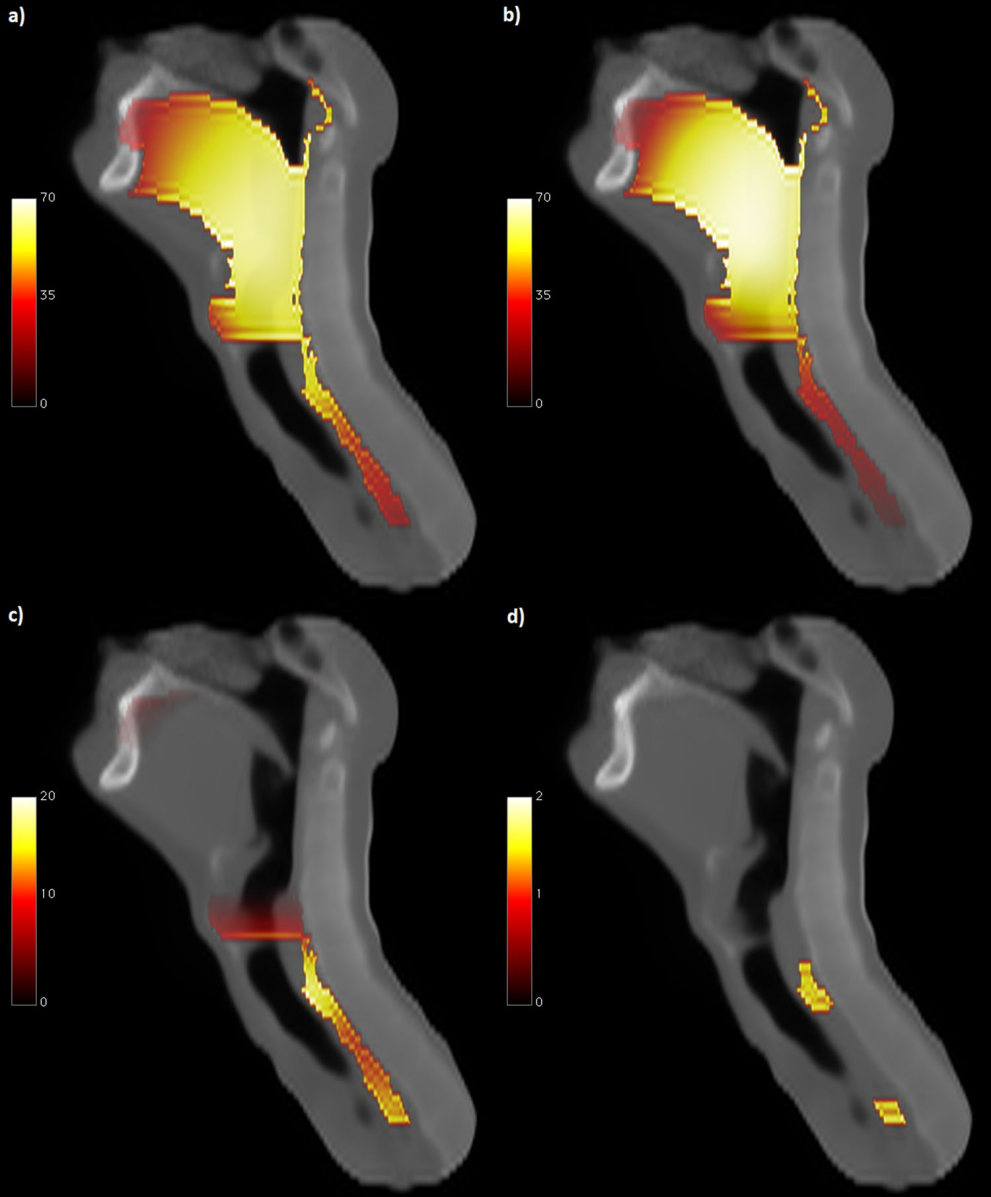
Dose distribution differences. Sagittal view of: mean dose map (Gy) for patients (**a**) who experienced radiation-induced acute dysphagia and (**b**) who did not; (**c**) corresponding dose difference map; (**d**) subregions showing a statistically significant dose difference between groups (*p* < 0.05) according to TFCE permutation test (the color map represents -Log *p*).

**Figure 4:**
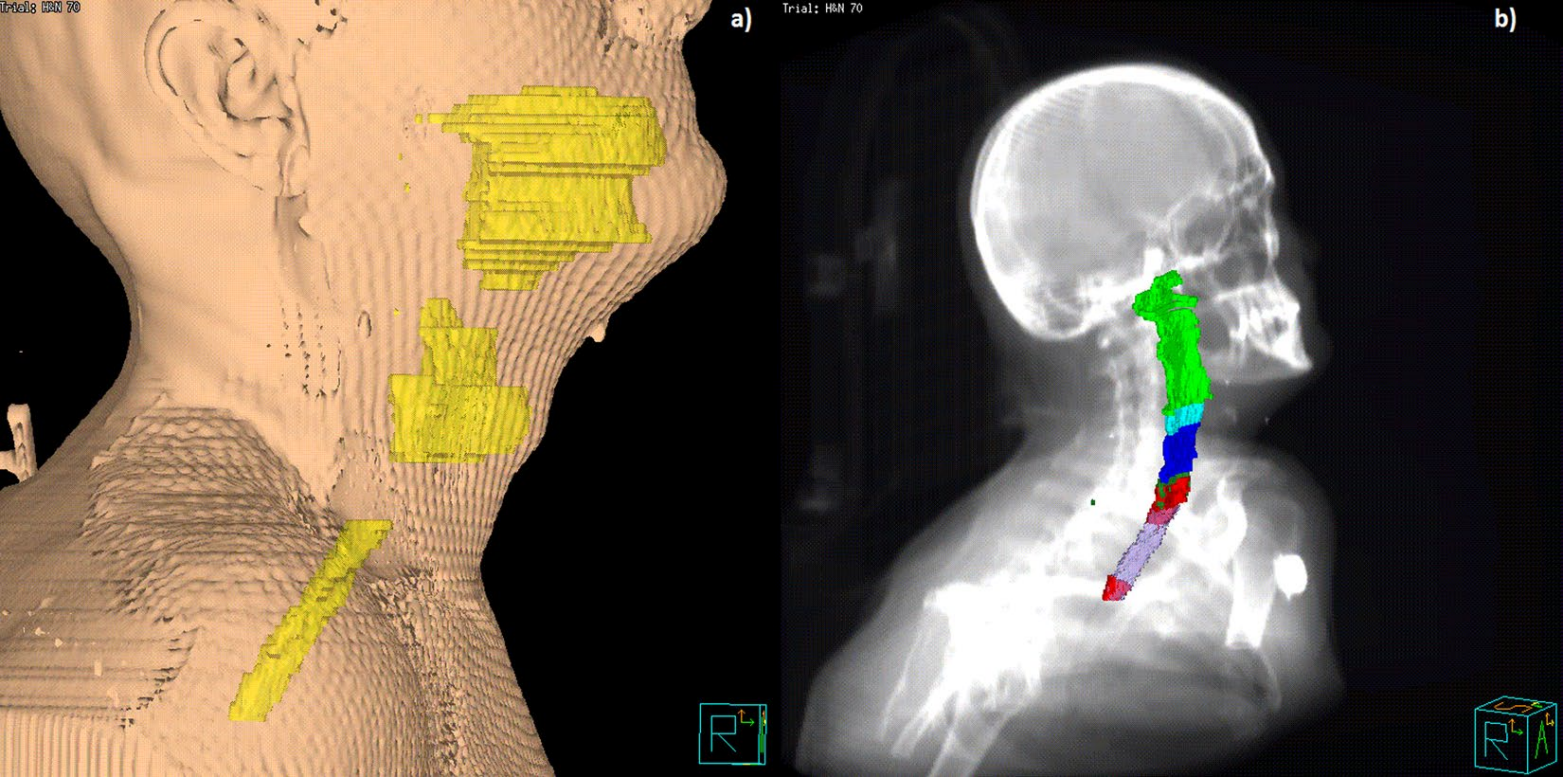
Volume rendering of head and neck structures in the common coordinate system: (**a**) 3D body rendering of the reference patient along with the three structures (yellow) used to derive the registration mask (oral cavity, larynx and esophagus); (**b**) 3D rendering of the superior (light green), middle (cyan) and inferior (blue) pharyngeal constrictor muscles, the cricopharyngeus muscle (forest green) and the cervical esophagus (violet), along with the *S*_0.05_ structure (red), superimposed on the Digital Reconstructed Radiograph.

**Figure 5:**
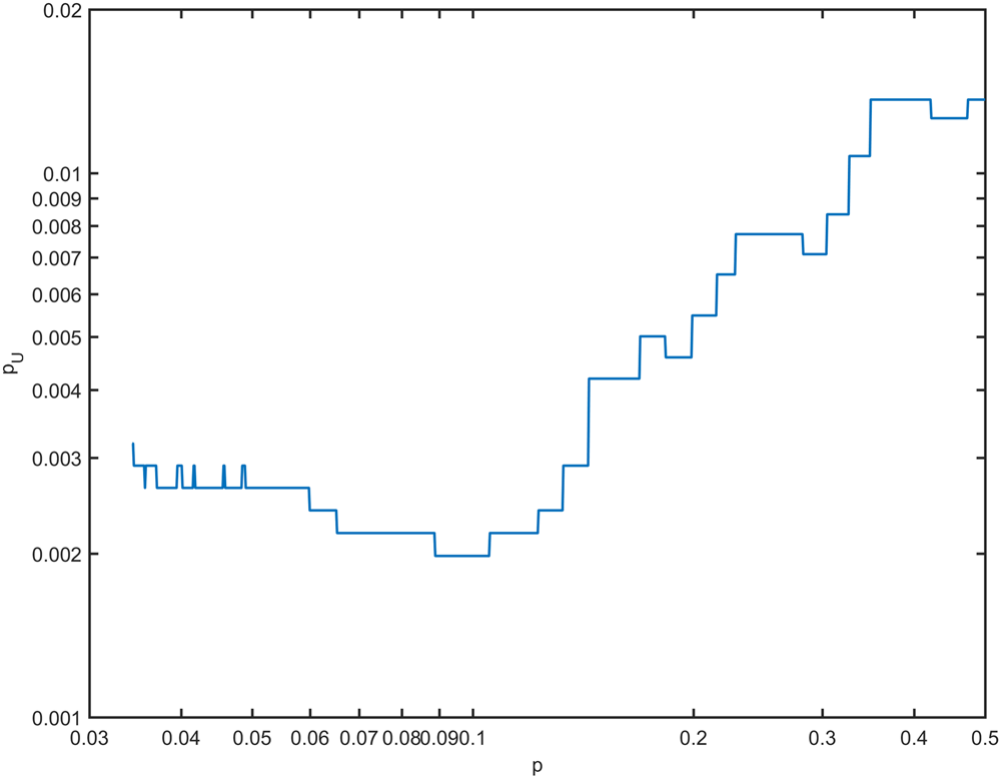
Discriminating power of mean dose in *S*_*p*_ subregions. Significance (*p*_*U*_), as evaluated by a Mann-Whitney *U* test, of the mean dose differences on *S*_*p*_ between patients who developed toxicity and who did not.

A significantly higher dose was delivered to RIAD patients, given a *p* = 0.038 value of the *T*_max_ statistic. The *S*_0.05_ overall volume of 5.1 cm^3^ was located in two voxel-clusters in correspondence of the cricopharyngeus muscle and cervical esophagus (Figs 3 and 4). The area under the ROC curve (AUC) from the mean dose on *S*_0.05_ (*D*_*m,i*_) resulted to be 0.81 (95% CI: [0.67, 0.95]). On RIAD patients, *D*_*m,i*_ ranged from 38 Gy to 58 Gy (median = 48 Gy) while on non-RIAD patients, *D*_*m,i*_ ranged from 10 Gy to 57 Gy (median = 33 Gy); at a Mann-Whitney U test the two distributions were significantly different (*p* = 0.003). Interestingly, the significance *p*_*U*_ of the mean dose differences between RIAD and non-RIAD patients within *S*_*p*_ subregions showed a global maximum (i.e. *p*_*U*_ attains a minimum) roughly in correspondence of *p* = 0.05 (Fig. 5).

The *p*-value computed according to Kolmogorov-Smirnov test on $\{{D}_{m,i}|i\in {RIAD}\}$ was 0.99, thus confirming that mean dose values in RIAD patients followed a Gaussian distribution. The first percentile of the distribution was thus estimated by equation (1) to be equal to 30.5 Gy.

**Figure 6:**
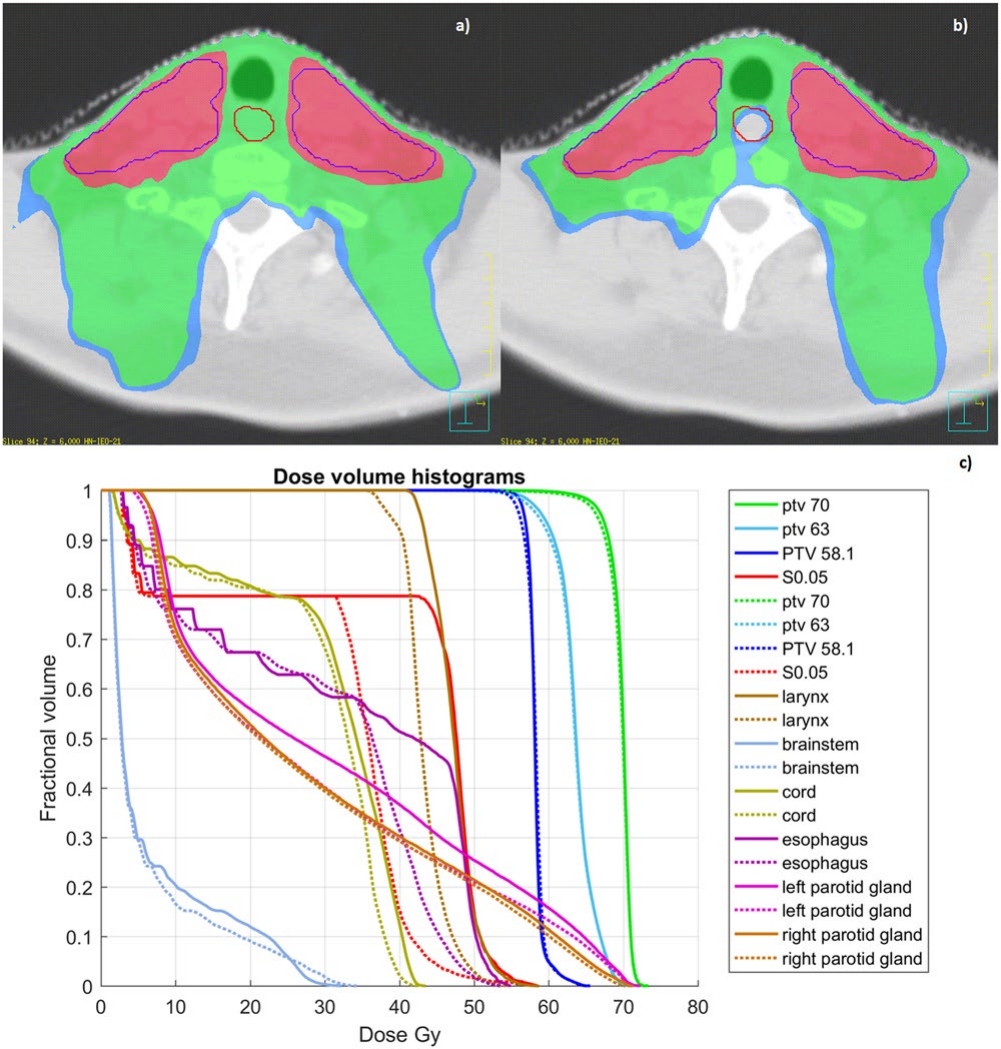
Treatment plan optimization. Plan_1_ (**a**) and Plan_2_ (**b**) dose distributions (the red line is the *S*_0.05_ structure) along with the comparative dose-volume histograms (**c**) for planning target volumes, organs at risk and the *S*_0.05_ structure (solid lines for Plan_1_ and dashed lines for Plan_2_). In the color wash, the blue, green and red regions correspond to 36 Gy, 38 Gy and 55.2 Gy dose levels, respectively.

The introduction of the *S*_0.05_ structure in Plan_2_ and the additional requirement in the optimization procedure that D_mean,*S*0.05_ does not exceed 30.5 Gy led to a large sparing of the *S*_0.05_ (D_mean,*S*0.05_: 30.4 Gy on Plan_2_ vs. 39.1 Gy on Plan_1_) at the cost of a negligible loss in the PTVs compared to Plan_1_ without jeopardizing the attainment of OAR objectives (Fig. 6).

## Discussion

We established a comprehensive pipeline for the application of a VB approach to explore and to exploit the relationship between local dose release and radiation induced morbidity (Fig. 1). In particular, we implemented a procedure to assess local dose differences potentially associated to the development of RIAD. The developed tool was applied to a cohort of HN cancer patients treated with definitive RT and the identified significant subregion was subsequently used for TP optimization purpose in a representative patient.

The technological RT developments, such as IMRT, have contributed to improve local tumor control in HN cancer patients, but also to reduce normal tissue doses, thus preventing radiation-induced side effects. Radiation induced toxicities in HN structures remain however the major dose-limiting factors and many survivors must cope with long-term effects of radiation treatments negatively impacting their quality of life. Besides radiation induced xerostomia, the swallowing dysfunction (i.e. dysphagia) is becoming one of the most relevant side effects of HN RT, more frequently observed in association with concomitant chemotherapy^12, 13^. Numbers of studies identified the DVH-parameters associated with both acute and late dysphagia in curative HN RT^11–14, 28–30^. Interestingly, acute dysphagia was a strong prognostic factor for late dysphagia^31^. However, most of those studies applied an organ-based approach that relies on identifying the multiple muscles and structures involved in the swallowing dysfunction, thus leading to a huge amount of dosimetric parameters and to the inherent well-known overfitting issue^11, 13^. In addition, the tiny and elusive swallowing related OARs exhibit a poor contrast on planning CTs (e.g. the cricopharyngeus and the three levels of pharyngeal constrictor muscles), hence posing concrete risks of structure misclassification. Indeed, the studies available in literature reported heterogeneous results concerning the dose parameters predictive for late or acute dysphagia.

It is our belief that a possible solution to this conundrum is represented by a reversed, i.e. holistic, approach to the radiation-induced toxicity analysis. Instead of splitting further and further the organs into their substructures, the VB approach allows for a blind identification of the involved regions, irrespective of their anatomical classification.

VB analyses have long been exploited in several neuroimaging frameworks, and the underlying philosophy has been recently introduced in the RT toxicity studies. The correlation between regional dose deposition and a toxicity outcome has been explored for a few organs, such as bladder, rectum and lungs^3–8^.

One of the major issues of the VB approach consists in the massive multiple comparison problem, which hinders a straightforward application of standard statistical tests, such as parametric (*t*) or non-parametric (Mann-Whitney) hypothesis tests. Solutions are available from the neuroimaging literature^32^; in this respect, we here adopted the non-parametric permutation inference coupled to the TFCE method^26^, which is particularly effective to enhance areas of signal that exhibit some spatial contiguity without relying on hard-threshold-based clustering.

The other central issue of the VB analysis concerns the inter-patient registration strategy adopted to anchor all the dose distributions to a common anatomical reference, which greatly impacts on the dose warping accuracy. In this study we implemented a multi-resolution version of the log-diffeomorphic demons approach^22^ which emerged as a highly performing scheme in the HN district according to Rigaud *et al*.^18, 19^. Indeed, the visual inspection of Fig. 2 as well as the Dice and MHD scores, reported in Table 1, indicates that the registration process was geometrically robust. Similarly, the DOO index shows that the method is accurate in dose warping.

The joint application of registration process and statistical mapping led to the identification of a regional dose-RIAD relationship. Interestingly, the highlighted statistically significant subregion *S*_0.05_ is located in correspondence of the cricopharyngeus muscle and cervical esophagus (Figs 3 d and 4b), with a median dose of 48 Gy in RIAD patients compared to the median dose of 33 Gy released to non-RIAD patients. The ROC analysis revealed a good prediction performance (AUC = 0.8) of the mean dose extracted from the subregion *S*_0.05_. Of note, Otter *et al*.^13^ found that mean dose to inferior pharyngeal constrictor muscles, immediately above the upper cluster in *S*_0.05_, is correlated with grade 3 acute dysphagia. Differently, the dose to the 2% of the superior pharyngeal constrictor was found to be a dosimetric predictor for the same endpoint by De Ruyck *et al*.^12^.

The evidence of a correlation between the dose delivered in a specific region of the swallowing-involved organs and the RIAD onset can be exploited to construct an avoidance criterion in the planning optimization strategy. The findings obtained by the statistical mapping analysis indeed allowed for an optimization of the TP procedure in a HN cancer patient external to the analyzed cohort. Furthermore, we demonstrated the practical feasibility of a *S*_0.05_ sparing plan (Plan_2_) within an IMRT framework (Fig. 6). In particular, the auto-planning engine coupled with the VMAT technique was applied to an oropharyngeal cancer patient with and without the *S*_0.05_ mean dose constraint. By adding the D_mean,*S*0.05_ <30.5 Gy to the standard OAR optimization goals for the HN cancer IMRT and with only one single optimization cycle, we succeeded to sharply decrease the mean dose to the *S*_0.05_ structure and even to improve the sparing of all the OARs involved as it can be observed in Fig. 6c.

Remarkably, the presented results were obtained thanks to the heterogeneity of dose distribution within the considered cohort. Should the doses to the subregion *S*_0.05_ be lowered by a dedicated TP scheme, it may be expected that the incidence and severity of RIAD would be accordingly reduced, thus allowing the proposed method to provide knowledge on milder grade of radiation induced toxicities.

In conclusion, the VB method applied to HN tumor patients treated with RT successfully allowed to identify HN regions correlated with radiation-induced morbidity and, as such, it might be included in the IMRT optimization strategy. Overall, the proposed approach may provide new insights into spatial signature of radiation sensitivity in a highly composite region such as the HN district. The practical relevance of the VB analysis in HN clinical TP optimization was also demonstrated.
